# Diagnostic performance of rapid antigen tests (RAT) for COVID-19 and factors associated with RAT-negative results among RT-PCR-positive individuals during Omicron BA.2, BA.5 and XBB.1 predominance

**DOI:** 10.1186/s12879-024-09408-8

**Published:** 2024-05-21

**Authors:** Celine Y. Tan, Kangwei Zeng, Lin Cui, Raymond T P Lin, Mark Chen

**Affiliations:** 1 National Preventive Medicine Residency Programme, National University Health System, Singapore, Singapore; 2 National Centre for Infectious Diseases, Singapore, Singapore; 3 Ministry of Health, Singapore, Singapore

**Keywords:** COVID-19, SARS-CoV-2, Rapid antigen test, Diagnostic performance, Sensitivity

## Abstract

**Background:**

While numerous studies have evaluated the real-world performance of rapid antigen tests (RATs), data on the effect of Omicron sublineages such as XBB and reinfections on RAT performance is limited. We assessed the performance of RATs and factors associated with RAT-negative results among individuals who tested SARS-CoV-2-positive by reverse transcription-polymerase chain reaction (RT-PCR).

**Methods:**

We conducted a retrospective study among Singapore residents who underwent testing for SARS-CoV-2 with RAT (Acon Flowflex or SD Biosensor) and RT-PCR in the same clinical encounter between 9 May 2022 and 21 November 2022. RT-PCR served as a reference standard for RAT performance. Logistic regression was used to estimate the odds ratios (OR) of factors associated with negative RAT results among RT-PCR-positive cases.

**Results:**

Of 8,620 clinical encounters analysed, 3,519 (40.8%) were SARS-CoV-2-positive on RT-PCR. Overall sensitivity and specificity of RAT was 84.6% (95% CI 83.3–85.7%) and 99.4% (95% CI 99.1–99.6%) respectively. Acon Flowflex consistently achieved higher sensitivity and specificity than SD Biosensor test kit. Among RT-PCR-positive cases, individuals who had a previous documented SARS-CoV-2 infection, coinfection with another respiratory pathogen or tested ≥ 6 days from symptom onset had higher odds of testing RAT-negative, but the associations were attenuated after adjustment for cycle threshold values (proxy for viral load). There was no significant difference in RAT performance between Omicron sublineages BA.2, BA.5 and XBB.1.

**Conclusion:**

Diagnostic performance of RAT was not affected by changes in predominant circulating Omicron sublineages. However, reinfection cases may be under ascertained by RAT. In individuals with a previous SARS-CoV-2 infection episode or symptom onset ≥ 6 days prior to testing, a confirmatory RT-PCR may be considered if there is high clinical suspicion.

**Supplementary Information:**

The online version contains supplementary material available at 10.1186/s12879-024-09408-8.

## Introduction

While the reverse transcription-polymerase chain reaction (RT-PCR) test remains the gold standard to detect SARS-CoV-2, it is time-consuming and costly, requiring trained laboratory personnel and dedicated facilities. Since the development of rapid antigen tests (RATs) for SARS-CoV-2 detection, they have served as an important diagnostic tool for COVID-19, being less costly and resource-intensive while allowing prompt detection to inform clinical management and public health decisions. In settings with ongoing community transmission, RATs may be used for diagnosis of COVID-19 among individuals who are symptomatic or had recent exposure to SARS-CoV-2. Self-testing with RATs can also be used for screening purposes among individuals without symptoms or known exposure to SARS-CoV-2, irrespective of the level of community transmission [1]. With RATs becoming ubiquitous, it is important that there are clear instructions and training of healthcare personnel in using RATs for valid sample collection and reliable results.

In Singapore, RATs have been the main testing modality for SARS-CoV-2 since early 2022 [2], and diagnosis of COVID-19 based on RAT is used to inform clinical management and other policy decisions such as easing of safe distancing measures and recommended timings of COVID-19 vaccinations [3, 4]. Furthermore, there has been increasing use of self-testing with RATs, with frequent distribution exercises of RAT kits by the government to each household [5]. It is thus important to monitor and evaluate the performance of RATs.

Numerous studies have evaluated the real-world performance of RATs in various settings, with one 2022 meta-analysis which included over 150 studies reporting the sensitivity and specificity among symptomatic patients to be 73.0% and 99.1% respectively [6]. Viral load has also been found to be the most important factor in determining RAT sensitivity, based on a systematic review which included 83 studies [7]. With the genesis of new SARS-CoV-2 variants with enhanced fitness driven by mutations in the spike protein responsible for target recognition and cellular entry [8], studies have also assessed RAT performance over time. A United Kingdom study which analysed data collected between November 2020 and March 2022 found that RATs were able to detect most SARS-CoV-2 infections throughout vaccine roll-out and across the pre-alpha or Alpha, Delta, and Omicron BA.1/BA.2 variants [9]. 

However, the landscape of COVID-19 has evolved rapidly, with the emergence of numerous Omicron subvariants including XBB and its descendent lineages, and a high rate of reinfections [10, 11]. A Japanese study which evaluated seven kinds of RATs found that all showed similar sensitivity to the Omicron subvariants BA.5, BA.2.75, BF.7, XBB.1, and BQ.1.1 [12]. Data on the effect of reinfection on the performance of RATs remain limited. A Czechia study reported that RAT sensitivity for Omicron cases with a previous infection was 79.2% (95% confidence interval [CI] 77.8–80.5%) compared with 81.9% (95% CI 81.3–82.5%) for those without a confirmed previous infection, but the study was conducted during the early Omicron period with data up to February 2022 [13]. 

In this study, we aimed to assess the performance of RATs compared with RT-PCR as a gold standard, and identify factors associated with negative RAT results among cases who tested SARS-CoV-2 positive by RT-PCR during the period of Omicron BA.2, BA.5 and XBB.1 predominance in Singapore.

## Methods

### Study design, setting, and participants

We conducted a retrospective study among individuals who were tested for SARS-CoV-2 using RAT and RT-PCR during the same clinical encounter between 9 May 2022 and 21 November 2022. Under the Acute Respiratory Infection (ARI) surveillance programme at 11 polyclinics in Singapore which served as sentinel outpatient primary care sites, individuals presenting with acute respiratory symptoms of cough, runny nose, sore throat and/or fever were randomly selected and offered to join the surveillance programme by their clinician. Individuals who were willing to participate then underwent testing with a healthcare-administered RAT and RT-PCR. We evaluated the two RAT kits predominantly used in the sentinel clinics, Flowflex SARS-CoV-2 Antigen Rapid Test (Acon Laboratories) and STANDARD Q COVID-19 Ag Home Test (SD Biosensor).

Specimens for RT-PCR were tested using the BioFire® Respiratory 2.1 (RP2.1) Panel (bioMérieux, France), a multiplex PCR which allows the simultaneous detection of multiple viral and bacterial respiratory organisms, including SARS-CoV-2 [14]. RT-PCR-positive cases were those which were SARS-CoV-2-positive on RP2.1 panel. The SARS-CoV-2-positive specimens were then tested using TaqPath™ COVID-19 Combo Kit (ThermoFisher, USA) and selected for whole genome sequencing (WGS) based on the cycle threshold (Ct) and S-gene target failure (SGTF) status. 40 PCR cycles were run as recommended by the manufacturer, and specimens with Ct value < 30 could be selected for WGS.

WGS was conducted on specimens using the ARTIC nCoV-2019 amplicon panel (Integrated DNA Technologies) and the Nextera XT DNA Library Preparation Kit (Illumina) on the Illumina MiSeq platform in accordance with the manufacturers’ instructions. The viral genome sequences were assembled from raw data by in-house pipelines and the viral lineages were determined by Pangolin.

### Data sources and variables of interest

Data were collected from databases maintained by the National Public Health Laboratory and Ministry of Health, Singapore. Paired RAT and RT-PCR results for SARS-CoV-2, as well as age, sex, ethnicity, brand of RAT kit, presence of co-infection with other viral respiratory pathogen(s), clinic attended, vaccination status at time of infection, previous infection status, days between symptom onset and sample collection and SARS-CoV-2 lineage (if sequenced) were collected. Ct value was used as a proxy indicator of viral load, based on the lowest Ct value of the three targets in the TaqPath™ COVID-19 Combo Kit assay. Swab collection site for RT-PCR (nasopharyngeal, nasal, oropharyngeal and mid-turbinate, or throat) was recorded by healthcare staff and collected, but anatomical collection site of RAT was not known. Completion of the primary vaccination series was defined as two doses of Pfizer-BioNTech/Comirnaty or Moderna-Spikevax, three doses of Sinovac-CoronaVac or Sinopharm BBIBP-CorV, or two doses of non-mRNA vaccines approved under the World Health Organization (WHO) Emergency Use Listing besides Sinovac-CoronaVac and Sinopharm BBIBP-CorV [15]. Individuals who received additional vaccine doses after the primary vaccination series were considered boosted.

Those who had a SARS-CoV-2 infection notified to the Ministry of Health at least 90 days before the date of study inclusion were considered to have a previous documented infection [16]. In January 2022, Omicron overtook Delta as the predominant strain in Singapore and comprised over 91% of local cases which were sequenced [17]. Hence, individuals with a documented infection episode before 1 January 2022 were considered to have a previous pre-Omicron infection, while those with a previous documented infection from 1 January 2022 were assumed to have an Omicron infection. Individuals who tested RT-PCR-positive during the study period and did not have a previous documented infection were considered first infections.

### Statistical analyses

RAT sensitivity, specificity, negative predictive value, and positive predictive value were calculated using BioFire RP2.1 Panel RT-PCR as the reference standard, with 95% CI calculated. Logistic regression models were used to estimate odds ratios (OR) of factors with negative RAT results among those who were SARS-CoV-2-positive by RT-PCR. Two multivariable logistic regressions were constructed. Model 1 adjusted for demographics, COVID-19 vaccination status, RAT brand, presence of co-infection with other respiratory pathogens, previous known SARS-CoV-2 infection, days between symptom onset and sample collection, SARS-CoV-2 lineage, PCR sample type and clinic visited, while Model 2 adjusted for factors in Model 1 and Ct value, to elucidate the effect of Ct values on the associations. Adjusted ORs of Models 1 and 2 were labelled aOR1 and aOR2 respectively. All data analysis was performed using Stata version 15.0 (StataCorp, College Station, TX, USA). A p-value of <0.05 was considered statistically significant.

### Ethics approval

The study received ethics approval from the NHG Domain Specific Review Board (DSRB Ref: 2023/00131) with waiver of informed consent.

## Results

### Diagnostic performance of RAT for COVID-19

A total of 8,620 clinical encounters were analysed, comprising 3,519 (40.8%) SARS-CoV-2-positive and 5,101 (59.2%) SARS-CoV-2-negative samples by RT-PCR based on the BioFire RP2.1 Panel (Table 1). 2,976 of RT-PCR-positive cases tested positive on RAT, achieving an overall sensitivity of 84.6% (95% CI 83.3–85.7%), while 5,068 of RT-PCR-negative cases tested negative on RAT, giving a specificity of 99.4% (95% CI 99.1–99.6%). Positive and negative predictive values were 98.9% (95% CI 98.5–99.2%) and 90.3% (95% CI 89.5–91.1%) respectively.

**Table 1:** Diagnostic performance of rapid antigen tests

	RT-PCR-positive	RT-PCR-negative	Total	
**RAT-positive**	2,976	33	3,009	Positive predictive value = 98.9%
**RAT-negative**	543	5,068	5,611	Negative predictive value = 90.3%
**Total**	3,519	5,101	8,620	
	Sensitivity = 84.6%	Specificity = 99.4%		

**Fig. 1:**
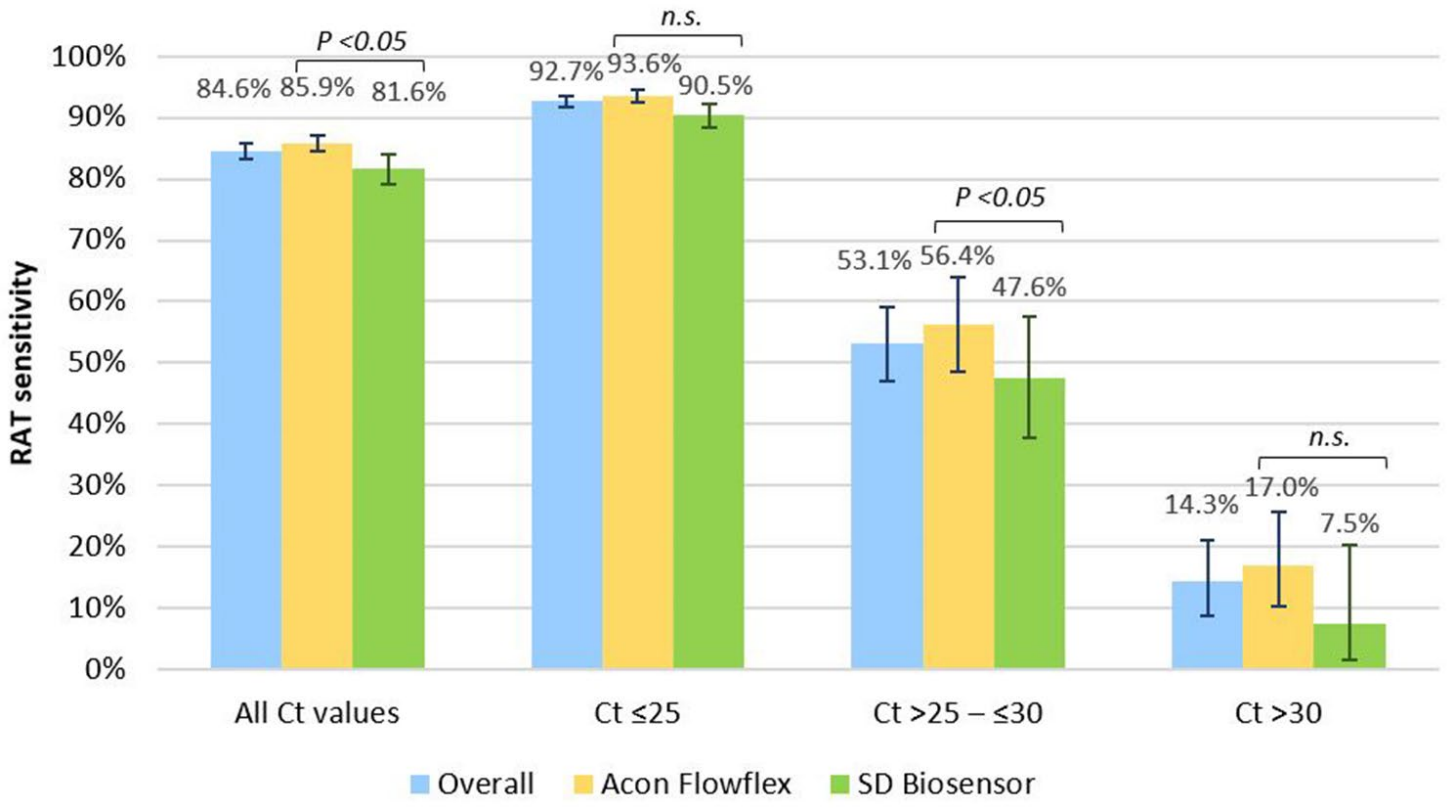
RAT sensitivity by cycle threshold (Ct) value & brand of RAT kit

By brand of RAT kits, Acon Flowflex achieved a sensitivity of 85.9% (95% CI 84.4–87.2%) and specificity of 99.4% (95% CI 99.0–99.6%), while sensitivity and specificity of SD Biosensor were 81.6% (95% CI 79.1–83.9%) and 99.4% (95% CI 98.8–99.7%) respectively (Fig. 1). At Ct values ≤ 25, sensitivity was 92.7% (95% CI 91.7–93.6%) overall, 93.6% (95% CI 92.5–94.6%) for Acon Flowflex and 90.5% (95% CI 88.4–92.3%) for SD Biosensor. At Ct values > 25 to ≤ 30, sensitivity decreased to 53.1% (95% CI 47.0–59.1%) overall, 56.4% (95% CI 48.6–63.9%) for Acon Flowflex and 47.6% (95% CI 37.8–57.6%) for SD Biosensor. RAT sensitivity further decreased to 14.3% (95% CI 8.9–21.2%) overall at Ct values > 30.

**Fig. 2:**
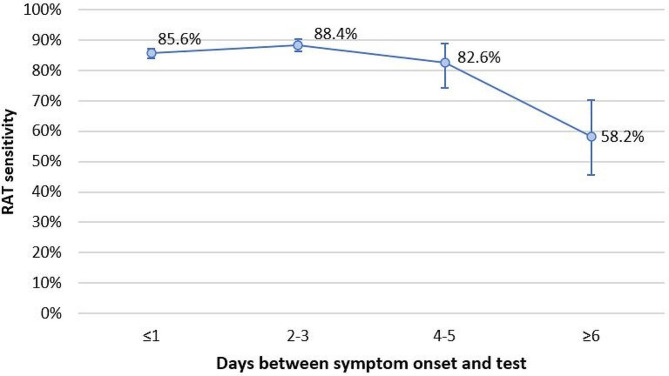
RAT sensitivity by days between symptom onset and test

Stratification by number of days between symptom onset and test collection showed that RAT sensitivity peaked at 88.4% (95% CI 86.2–90.4%) 2–3 days after symptom onset and declined to 58.2% (95% CI 45.5–70.2%) when the test was administered ≥ 6 days from symptom onset (Fig. 2).

Among those who were RT-PCR-positive, 320 (9.1%) had a previous pre-Omicron or Omicron infection. RAT sensitivity was 75.6% (95% CI 70.5–80.2%) for reinfection cases, compared with 85.5% (95% CI 84.2–86.7%) for first infections.

### Factors associated with RAT-negative results among RT-PCR-positive cases

The odds of a negative RAT result increased substantially with increasing Ct values. Compared with Ct < 20, Ct > 20 and ≤ 25 had 3.26 times (95% CI 2.38–4.46) higher odds of producing a negative RAT result, and Ct > 25 to ≤ 30 had an adjusted OR of 16.11 (95% CI 11.15–23.28) (Table 2).

**Table 2:** Association of factors with negative antigen rapid tests among RT-PCR-positive cases

	All RT-PCR-positive, *n* (%) *N* = 3,519	RAT-positive, *n* (%) *n* = 2,976	RAT-negative, *n* (%) *n* = 543	Univariate model		Model 1^a^		Model 2^b^	
				Unadjusted OR (95% CI)	*P*-value	Adjusted OR (aOR1) (95% CI)	*P*-value	Adjusted OR (aOR2) (95% CI)	*P*-value
**Age (years)**									
< 20	313 (8.9)	266 (8.9)	47 (8.7)	0.79 (0.56–1.11)	0.169	0.91 (0.60–1.37)	0.654	1.13 (0.69–1.84)	0.633
20–39	1,221 (34.7)	997 (33.5)	224 (41.3)	1 [ref]	-	1 [ref]	-	1 [ref]	-
40–59	1,201 (34.1)	1,019 (34.2)	182 (33.5)	0.79 (0.64–0.98)	0.036	0.88 (0.69–1.13)	0.312	0.96 (0.71–1.30)	0.802
≥ 60	784 (22.3)	694 (23.3)	90 (16.6)	0.58 (0.44–0.75)	< 0.001	0.58 (0.42–0.79)	< 0.001	0.57 (0.40–0.82)	0.003
**Sex**									
Male	1,952 (55.5)	1,640 (55.1)	312 (57.4)	1 [ref]	-	1 [ref]	-	1 [ref]	-
Female	1,567 (44.5)	1,336 (44.9)	231 (42.6)	0.91 (0.76–1.09)	0.311	0.90 (0.73–1.12)	0.356	0.93 (0.72–1.20)	0.590
**Ethnicity**									
Chinese	2,388 (67.9)	2,028 (68.2)	360 (66.3)	1 [ref]	-	1 [ref]	-	1 [ref]	-
Malay	746 (21.2)	627 (21.1)	119 (21.9)	1.07 (0.85–1.34)	0.562	0.90 (0.68–1.18)	0.435	1.05 (0.76–1.45)	0.779
Indian	297 (8.4)	244 (8.2)	53 (9.8)	1.22 (0.89–1.68)	0.213	1.23 (0.85–1.77)	0.277	1.30 (0.84–2.00)	0.234
Others	88 (2.5)	77 (2.6)	11 (2.0)	0.80 (0.42–1.53)	0.507	0.45 (0.19–1.10)	0.079	0.31 (0.10–0.97)	0.045
**Vaccination status** ^**c**^ ** at time of infection**									
Unvaccinated / partially vaccinated	23 (0.7)	20 (0.7)	3 (0.6)	1.10 (0.30–4.05)	0.883	0.62 (0.07–5.28)	0.662	0.61 (0.04–8.96)	0.720
Completed primary series	166 (4.8)	147 (4.9)	20 (3.7)	1 [ref]	-	1 [ref]	-	1 [ref]	-
Boosted	3,329 (94.6)	2,809 (94.4)	520 (95.8)	1.36 (0.84–2.19)	0.205	1.91 (1.09–3.35)	0.022	3.85 (1.74–8.52)	0.001
**RAT kit**									
Acon Flowflex	2,461 (69.9)	2,113 (71.0)	348 (64.1)	1 [ref]	-	1 [ref]	-	1 [ref]	-
SD Biosensor	1,058 (30.1)	863 (29.0)	195 (35.9)	1.37 (1.13–1.66)	0.001	1.49 (1.15–1.94)	0.003	1.58 (1.15–2.15)	0.004
**Co-infection with another respiratory pathogen**									
No	3,335 (94.8)	2,854 (95.9)	481 (88.6)	1 [ref]	-	1 [ref]	-	1 [ref]	-
Yes	184 (5.2)	122 (4.1)	62 (11.4)	3.02 (2.19–4.16)	< 0.001	2.03 (1.34–3.07)	0.001	1.05 (0.59–1.88)	0.859
**Previous documented infection**									
No previous infection	3,199 (90.9)	2,734 (91.9)	465 (85.6)	1 [ref]	-	1 [ref]	-	1 [ref]	-
Previous pre-Omicron infection	115 (3.3)	81 (2.7)	34 (6.3)	2.47 (1.63–3.73)	< 0.001	3.46 (2.14–5.59)	< 0.001	1.80 (0.98–3.28)	0.056
Previous Omicron infection	205 (5.8)	161 (5.4)	44 (8.1)	1.61 (1.14–2.27)	0.007	2.14 (1.37–3.32)	0.001	0.70 (0.39–1.26)	0.234
**Days between symptom onset & test**									
≤ 1	2,146 (61.0)	1,839 (61.7)	307 (56.5)	1 [ref]		1 [ref]		1 [ref]	
2–3	916 (26.0)	810 (27.3)	106 (19.4)	0.78 (0.62–0.99)	0.043	0.67 (0.52–0.86)	0.002	0.79 (0.58–1.06)	0.113
4–5	115 (3.3)	95 (3.2)	20 (3.7)	1.26 (0.77–2.07)	0.36	1.24 (0.72–2.11)	0.438	1.51 (0.82–2.79)	0.182
≥ 6	67 (1.9)	39 (1.3)	29 (5.3)	4.30 (2.61–7.09)	< 0.001	4.02 (2.29–7.08)	< 0.001	2.25 (1.09–4.63)	0.028
Not available	275 (7.8)	193 (6.5)	82 (15.0)	-	-	-	-	-	-
**Lineage**									
BA.2	373 (10.6)	355 (11.9)	18 (3.3)	1 [ref]	-	1 [ref]	-	1 [ref]	-
BA.2.12.1	24 (0.7)	24 (0.8)	0	NA^f^	-	NA^f^	-	NA^f^	-
BA.2.7X^d^	77 (2.2)	74 (2.5)	3 (0.6)	0.80 (0.23–2.78)	0.725	0.59 (0.16–2.09)	0.410	0.47 (0.13–1.76)	0.266
BA.4	26 (0.7)	25 (0.8)	1 (0.2)	0.79 (0.10–6.15)	0.821	0.74 (0.09–5.84)	0.773	0.69 (0.09–5.53)	0.725
BA.5	831 (23.6)	765 (25.7)	66 (12.1)	1.70 (1.00–2.91)	0.052	1.51 (0.87–2.61)	0.145	1.43 (0.81–2.53)	0.220
XBB and descendent lineages^e^	359 (10.2)	338 (11.4)	21 (3.9)	1.23 (0.64–2.34)	0.538	0.79 (0.39–1.58)	0.502	1.02 (0.50–2.07)	0.953
Others	78 (2.2)	73 (2.5)	5 (0.9)	1.35 (0.49–3.75)	0.564	1.43 (0.50–4.07)	0.504	1.76 (0.61–5.08)	0.296
Not available	1,751 (49.8)	1,322 (44.4)	429 (79.1)	6.40 (3.94–10.40)	< 0.001	4.73 (2.88–7.79)	< 0.001	1.57 (0.92–2.68)	0.098
**Ct value**									
≤ 20	2,146 (61.0)	2,044 (68.7)	102 (18.8)	1 [ref]	-	-	-	1 [ref]	-
> 20 to ≤ 25	819 (23.3)	704 (23.7)	115 (21.2)	3.27 (2.47–4.33)	< 0.001	-	-	3.26 (2.38–4.46)	< 0.001
> 25 to ≤ 30	277 (7.9)	147 (4.9)	130 (23.9)	17.72 (13.02–24.13)	< 0.001	-	-	16.11 (11.15–23.28)	< 0.001
> 30	140 (4.0)	20 (0.7)	120 (22.1)	120.24 (71.95–200.92)	< 0.001	-	-	123.51 (66.71–228.68)	< 0.001
Not available	137 (3.9)	61 (2.0)	76 (14.0)	-	-	-	-	-	-

Ct = cycle threshold; OR = odds ratio; CI = confidence interval; RAT = rapid antigen test; ref = reference group

^a^ Model 1 adjusted for demographic factors, vaccination status, RAT brand, co-infection, previous known infection, days between onset and sample collection, PCR sample type, SARS-CoV-2 lineage and clinic visited

^b^ Model 2 adjusted for factors in Model 1 and Ct value

^c^ Primary series = 2 doses of Pfizer-BioNTech/Comirnaty or Moderna-Spikevax, 3 doses of Sinovac-CoronaVac or Sinopharm BBIBP-CorV, or 2 doses of non-mRNA vaccines approved under the WHO Emergency Use Listing besides Sinovac-CoronaVac and Sinopharm BBIBP-CorV; booster dose = additional vaccine dose received after primary series

^d^ Includes BA.2.75, BA.2.75.1, BA.2.75.2, BA.2.76, BA.2.78 and BA.2.79

^e^ Includes XBB, XBB.1, XBB.1.1 and XBB.2

^f^ Not applicable as all observations were RAT-positive

The predominant circulating SARS-CoV-2 variants during the study period were Omicron BA.2, BA.5 and XBB.1. On logistic regression with Omicron BA.2 as the reference group, there was no significant association between the various Omicron sublineages and discordant RAT results among RT-PCR-positive cases (Table 2).

Compared with Acon Flowflex test kit, SD Biosensor test kit was associated with higher odds of a negative RAT result, even after adjustment for all covariates (aOR2 1.58 [95% CI 1.15–2.15]). Individuals who received booster doses had higher odds of testing RAT-negative (aOR2 3.85 [95% CI 1.74–8.52]) than those who only completed the primary vaccination series.

Having a previous documented SARS-CoV-2 infection with pre-Omicron variant (aOR1 3.46 [95% CI 2.14–5.59]) or Omicron variant (aOR1 2.14 [95% CI 1.37–3.32]), or a coinfection with another respiratory pathogen (aOR1 2.03 [95% CI 1.34–3.07] were significantly associated with a negative RAT result on adjustment for covariates except Ct value (model 1). However, these associations were substantially attenuated and no longer statistically significant after further adjustment for Ct value (model 2).

Compared with testing within 1 day of symptom onset, individuals who were tested 6 days or more from symptom onset had higher odds of testing RAT-negative (aOR1 4.02 [95% CI 2.29–7.08], while those tested 2–3 days after symptom onset had lower odds of testing RAT-negative (aOR1 0.67 [95% CI 0.52–0.86]. These associations were also attenuated after adjusting for Ct value, with only ≥ 6 days between symptom onset and test administration remaining significant (aOR2 2.25 [95% CI 1.09–4.63]).

## Discussion

While both Acon Flowflex and SD Biosensor achieved the minimum criteria set by the WHO of ≥ 80% sensitivity and ≥ 97% specificity, we found that Acon Flowflex consistently performed better than SD Biosensor, consistent with similar previous studies [18, 19]. Our sensitivity estimates of 93.6% for Acon Flowflex and 90.5% for SD Biosensor at Ct values ≤ 25 were comparable with a German study evaluating diagnostic tests using a common panel of SARS-CoV-2 specimens, which reported sensitivities of 94.1% and 88.9% for Acon Flowflex and SD Biosensor respectively [18]. With increasing Ct values, there was a substantial decrease in RAT sensitivity and increase in odds of testing RAT-negative among RT-PCR-positive cases, consistent with systematic reviews that viral load is the most important factor influencing sensitivity [7, 20]. 

RAT performance did not significantly change between Omicron sublineages BA.2, BA.5 and XBB.1. This is aligned with current knowledge given that RATs target the nucleocapsid protein [21], while majority of mutations in SARS-CoV-2 variants of concern (VOC) occur in the spike protein [22]. These findings reassure us that the performance of RAT is not affected by the emergence of new variants and sublineages.

We found a marked decline in RAT sensitivity when the test was administered ≥ 6 days from symptom onset, and this association was mainly mediated by higher Ct values suggestive of lower viral load. This was congruent with a Brazilian study which found that greatest sensitivity of the antigen test was observed when the test was performed within 5 days of symptom onset [23], suggestive that viral load markedly decreases 5 days after symptom onset.

We also observed that individuals who were tested 2–3 days after symptom onset had lower odds of having a false negative RAT result compared with those tested within 1 day of symptom onset. A study conducted in Switzerland similarly found that sensitivity of the rapid test peaked at 2 days post onset of symptoms [24]. We further showed that the association was mediated by viral loads (using Ct value as a proxy marker), aligned with previous studies which reported peak viral loads in upper respiratory specimens around 3 days from symptom onset [25, 26]. Individuals with an initial negative RAT result may consider repeating the test 1–2 days after symptom onset or obtaining a RT-PCR test.

Of note, RAT sensitivity was 75.6% for known reinfection cases, around 10% less compared to first infections and below the WHO minimum criteria. Our results also showed that the association between having a previous pre-Omicron or Omicron infection and a negative RAT result was similarly mediated by viral loads. This is consistent with previous studies which found that infection-induced immunity was associated with shorter duration of viral shedding and lower viral loads [27]. In settings where RAT is the main testing modality, there may be under-detection of reinfection cases. This is relevant if clinical decisions such as commencement of oral antiviral treatment are made based on RAT results, and a confirmatory RT-PCR may be considered if there is high clinical suspicion. Under-ascertainment of reinfection cases from reduced RAT sensitivity should also be considered in the formulation of public health policies and COVID-19 control measures. Furthermore, RAT sensitivity is likely to decline as the seroprevalence among the population and proportion of reinfection cases continue to increase, thus the performance of RATs should be continuously evaluated.

Individuals who received booster doses were found to have higher odds of testing RAT-negative than those who only completed the primary vaccine series, even after adjustment for viral loads. A study conducted in Germany also observed that the antigen test was potentially less sensitive for samples with medium (20–30) Ct values in vaccinated than unvaccinated cases [28]. This may suggest differences in RAT performance by vaccination status, and further studies will be required to evaluate this association.

The strengths of this study include the use of comprehensive data collected under a surveillance programme, and paired sample collections in the same clinical encounter. However, there are also several limitations. First, we did not have data on the anatomical collection site for RAT specimen, which has been found to be a factor affecting RAT sensitivity [7]. RAT performance could also be affected by variable swab techniques between different healthcare workers, although we attempted to mitigate this by adjusting for the clinic which the patient had presented to in the logistic regression models. Data on the comorbidities of participants were also not available, hence we were unable to assess the effect of factors such as immunosuppression which may affect RAT performance. Second, the classification of previous infection status may not be precise due to under-ascertainment of mild or asymptomatic cases who did not present to healthcare facilities. Third, Ct value was used as a proxy indicator for viral load but is not a direct marker as it is affected by factors related to sample collection and processing and performance of the RT-PCR assay [29]. To mitigate this, all RT-PCR tests in this study were conducted in a single laboratory with the same assay, and specimen type was included as a covariate in the multivariable logistic regression model. Fourth, as the study only included symptomatic individuals who were selected for ARI surveillance, the results may not be generalisable to asymptomatic cases.

The results of our study can help to inform testing strategies to increase RAT sensitivity among symptomatic patients, such as the choice of test kits and testing within 5 days of symptom onset. While RAT performance did not significantly change between Omicron sublineages BA.2, BA.5 and XBB.1, we highlight that reinfections are less likely to be picked up on RAT, which can contribute to under-ascertainment of reinfection cases. The diagnostic performance of RATs may thus be periodically evaluated as the proportion of reinfection cases increases and the SARS-CoV-2 virus continues to mutate over time.

### Electronic supplementary material

Below is the link to the electronic supplementary material.

Supplementary Material 1

## Data Availability

The datasets analysed with individual-level information are not publicly available due to personal data protection.
