# Supplementary material for: Diagnostic performance of rapid antigen tests (RAT) for COVID-19 and factors associated with RAT-negative results among RT-PCR-positive individuals during Omicron BA.2, BA.5 and XBB.1 predominance
Source: BMC Infect Dis. 2024 May 21;24:504. doi: 10.1186/s12879-024-09408-8 (PMC11107039; doi:10.1186/s12879-024-09408-8)
Supplement: Supplementary file 1 — Supplementary Material 1 [file 12879_2024_9408_MOESM1_ESM.docx]

**SUPPLEMENTARY MATERIAL**

Table S1. Distribution of RT-PCR specimen types among RT-PCR-positive cases

| **RT-PCR specimen type** | **No. (%)** |
| --- | --- |
| Nasopharyngeal | 139 (3.9) |
| Nasal | 796 (22.6) |
| Oropharyngeal-mid turbinate | 2,468 (70.1) |
| Throat | 20 (0.6) |
| Not available | 96 (2.7) |

Table S2. Characteristics of false-positive cases (RAT-positive and RT-PCR-negative)

|  | **No. (%)** |
| --- | --- |
| **Age (years)** |  |
| <20 | 3 (9.1%) |
| 20 – 39 | 18 (54.6%) |
| 40 – 59 | 7 (21.2%) |
| ≥60 | 5 (15.2%) |
| **Sex** |  |
| Male | 19 (57.6%) |
| Female | 14 (42.4%) |
| **Ethnicity** |  |
| Chinese | 24 (72.7%) |
| Malay | 3 (9.1%) |
| Indian | 6 (18.2%) |
| Others | 0 (0.0%) |
| **Vaccination status at time of infection** |  |
| Unvaccinated / partially vaccinated | 1 (3.0%) |
| Completed primary series | 3 (9.1%) |
| Boosted | 28 (84.9%) |
| **Co-infection with another respiratory pathogen** |  |
| No | 31 (93.9%) |
| Yes | 2 (6.1%) |
| **Previous documented infection** |  |
| No previous infection | 27 (81.8%) |
| Previous pre-Omicron infection | 1 (3.0%) |
| Previous Omicron infection | 5 (15.2%) |
